# RNase H As Gene Modifier, Driver of Evolution and Antiviral Defense

**DOI:** 10.3389/fmicb.2017.01745

**Published:** 2017-09-14

**Authors:** Karin Moelling, Felix Broecker, Giancarlo Russo, Shinichi Sunagawa

**Affiliations:** ^1^Institute of Medical Microbiology, University of Zurich Zurich, Switzerland; ^2^Max Planck Institute for Molecular Genetics Berlin, Germany; ^3^Department of Microbiology, Icahn School of Medicine at Mount Sinai, New York NY, United States; ^4^Functional Genomics Center Zurich, ETH Zurich/University of Zurich Zurich, Switzerland; ^5^Department of Biology, Institute of Microbiology, ETH Zurich Zurich, Switzerland

**Keywords:** RNase H, reverse transcriptase, retroviruses, (Retro)-transposons, ribozymes, evolution, antiviral defense, immune systems

## Abstract

Retroviral infections are ‘mini-symbiotic’ events supplying recipient cells with sequences for viral replication, including the reverse transcriptase (RT) and ribonuclease H (RNase H). These proteins and other viral or cellular sequences can provide novel cellular functions including immune defense mechanisms. Their high error rate renders RT-RNases H drivers of evolutionary innovation. Integrated retroviruses and the related transposable elements (TEs) have existed for at least 150 million years, constitute up to 80% of eukaryotic genomes and are also present in prokaryotes. Endogenous retroviruses regulate host genes, have provided novel genes including the syncytins that mediate maternal-fetal immune tolerance and can be experimentally rendered infectious again. The RT and the RNase H are among the most ancient and abundant protein folds. RNases H may have evolved from ribozymes, related to viroids, early in the RNA world, forming ribosomes, RNA replicases and polymerases. Basic RNA-binding peptides enhance ribozyme catalysis. RT and ribozymes or RNases H are present today in bacterial group II introns, the precedents of TEs. Thousands of unique RTs and RNases H are present in eukaryotes, bacteria, and viruses. These enzymes mediate viral and cellular replication and antiviral defense in eukaryotes and prokaryotes, splicing, R-loop resolvation, DNA repair. RNase H-like activities are also required for the activity of small regulatory RNAs. The retroviral replication components share striking similarities with the RNA-induced silencing complex (RISC), the prokaryotic CRISPR-Cas machinery, eukaryotic V(D)J recombination and interferon systems. Viruses supply antiviral defense tools to cellular organisms. TEs are the evolutionary origin of siRNA and miRNA genes that, through RISC, counteract detrimental activities of TEs and chromosomal instability. Moreover, piRNAs, implicated in transgenerational inheritance, suppress TEs in germ cells. Thus, virtually all known immune defense mechanisms against viruses, phages, TEs, and extracellular pathogens require RNase H-like enzymes. Analogous to the prokaryotic CRISPR-Cas anti-phage defense possibly originating from TEs termed casposons, endogenized retroviruses ERVs and amplified TEs can be regarded as related forms of inheritable immunity in eukaryotes. This survey suggests that RNase H-like activities of retroviruses, TEs, and phages, have built up innate and adaptive immune systems throughout all domains of life.

## RT and Rnase H of Retroviruses and Retrovirus-Like Elements

The discovery of the reverse transcriptase (RT), initially described as replication enzyme of retroviruses in 1970 (Baltimore, 1970; Temin and Mizutani, 1970), was so unexpected that it was awarded a Nobel prize in 1975. Shortly after the RT, the retroviral ribonuclease H (RNase H) was identified in retrovirus particles as essential component for the replication of viral RNA via an RNA-DNA hybrid intermediate to double stranded DNA (dsDNA) (Mölling et al., 1971; Hansen et al., 1988; Tisdale et al., 1991). Historically, the RNase H has often been considered as part of the RT in retroviruses. However, the enzyme has its proper role, impact on evolution and importance for the degradation of nucleic acids in various biological processes. Similarly, the RT is of much more general importance than just replicating retroviral genomes. One prominent example is the telomerase, the RT or TERT, that elongates chromosomal ends in embryonic tissue and stem cells. Here, RNase H activity is not involved, since the template RNA needs to be copied repeatedly. Both the RT and the RNase H are among the most abundant proteins on our planet (Ma et al., 2008; Caetano-Anollés et al., 2009; Majorek et al., 2014).

RNases H are also present in pararetroviruses such as hepatitis B viruses, cauliflower mosaic viruses that infect plants, the monkey-specific spuma or foamy viruses, or the sheep lentivirus Visna Maedi Virus. They all require an RNase H to cooperate with the RT. Pararetroviruses follow the same life cycle as retroviruses except that the replication intermediates packaged into the virion are at a different stage so that they contain dsDNA, not ssRNA (Flint et al., 2015).

**FIGURE 1:**
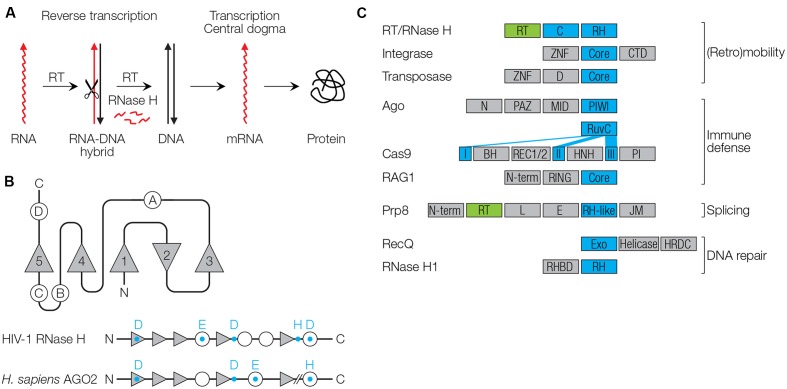
Function and structure of RNase H-like proteins. **(A)** Reverse transcriptase (RT), in concert with the RNase H catalyzes retroviral replication. **(B)** Conserved RNase H fold. β-sheets are shown as triangles (numbered 1–5), α-helices as circles (numbered A–D). Conserved catalytic residues are shown as blue letters. **(C)** Domain architectures of proteins with RNase H-like (RH) domains (blue) and RT domains (green). RT/RNase H (HIV-1): C, connection/tether/linker domain (Artymiuk et al., 1993). Integrase (HIV-1): ZNF, zinc finger domain; Core, catalytic core; CTD, C-terminal domain with DNA binding activity (Hindmarsh and Leis, 1999). Transposase, D, dimerization domain (Jiang et al., 2016). Argonaute (*Homo sapiens* AGO2): N, N-terminal domain; PAZ, PIWI/Argonaute/Zwille domain recognizing the 3′ end of small RNAs; MID, middle domain recognizing the 5′ end of small RNAs; PIWI, RNase H-like domain (Song et al., 2004). Cas9 (*Streptococcus pyogenes*): The RuvC domain with RNase H fold is separated into three parts (I–III); BH, bridge helix domain; REC1/2, recognition domains (REC1 binds the guide RNA); HNH, nuclease domain; PI, PAM-interacting domain (Jinek et al., 2014; Nishimasu et al., 2014). Rag-1 (*H. sapiens* RAG1): N-term, N-terminal domain with ubiquitin ligase activity; RING, Really Interesting New Gene domain with zinc finger motif; Core, catalytic core domain with endonuclease that also contains a zinc finger motif (Bassing et al., 2002; Yurchenko et al., 2003). Prp8 (*Saccharomyces cerevisiae*): N-term, N-terminal; L, linker; E, endonuclease-like; JM, Jab1/MPN (Nguyen et al., 2015). RecQ (*H. sapiens*, Werner Syndrome): Exo, 3′ to 5′ exonuclease domain; helicase and HRDC, RNase D C-terminal domain involved in DNA and protein interactions; NLS, nuclear localization signal (Oshima, 2000). RNase H1 (*H. sapiens*): RHBD, RNA/DNA hybrid binding domain (Nowotny et al., 2005).

Contrary to Francis Crick’s ‘central dogma’ from 1958 the RT, in concert with the RNase H, allows for the flow of information to occur from RNA to DNA. This was regarded as the reverse orientation, a historical view rooted in the discovery that DNA was the carrier of genetic information (**Figure 1A**). Reverse transcription of RNA occurring in concert with an RNase H is not restricted to retroviruses and pararetroviruses but is important also for retrotransposons that amplify via a ‘copy-and-paste’ mechanism such as the Ty elements of yeast. This mode of replication is closely related to that of retroviruses. However, there is no particle formation, release, and exogenous infection, since an envelope gene is missing. Genetic information is retrotransposed and can amplify within genomes.

An appreciation of the biological importance of RNA is increasing. RTs, as well as RNases H can exert crucial roles in the biogenesis and degradation of RNA molecules independently of each other. RNases H are involved whenever processing, trimming, or removal of genetic information is required – which happens as frequently as does the *de novo* synthesis of nucleic acid polymers. In theory, synthesis and degradation of nucleic acids should be in a well-balanced equilibrium. The RNase H-like structure is involved in numerous cleavage enzymes such as the retroviral integrase. The retroviral life cycle requires an integrase, which allows for inserting the DNA provirus into the cellular genome. Integrases adopt an RNase H-like core structure. Similarly, the ‘cut-and-paste’ replicative mechanism of transposable elements (TEs) also requires an integrase-like enzyme termed transposase (similarly with an RNase H fold), independently of an RT. The RT itself can also act independently of an RNase H, as in the case of telomerase, the enzyme that extends the ends of chromosomes. Telomerase depends on a short RNA molecule that is copied repeatedly – template degradation by an RNase H must not occur. In contrast, DNA-dependent DNA polymerases require RNases H for the removal of RNA primers after they have served their function, whereby the RNase H, in this case, is not fused to the polymerase as in retroviruses but is a separate molecule.

It came as a surprise when sequencing of the human genome revealed that almost 50% of its sequence is composed of retrovirus-like elements such as long and short interspersed nuclear elements (LINEs and SINEs), endogenous retroviruses (ERVs) often shortened to solitary LTRs, and Alu elements (a subclass of SINEs) that are common source of mutation in humans (Lander et al., 2001). Human ERVs (HERVs) populate the human genome and result from former germ line cell infections up to 150 Mio or more years ago.

The RNase H was first discovered in lysates of calf thymus, with unknown functions for a long time (Stein and Hausen, 1969). RNase H activity was also early described in the yeast *Saccharomyces cerevisiae*, where it was found to be involved in DNA replication (Karwan and Wintersberger, 1986). DNA replication requires RNA primers to initiate lagging strand DNA synthesis and their subsequent removal by the RNase H. The abundance of RT enzymes in bacteria, with 1021 different types identified, is surprising, yet their functions remain poorly understood (Simon and Zimmerly, 2008).

## RNase H in Various Species

RNases H are essential for degrading the RNA template during reverse transcription of the retroviral genome, thereby generating short RNA primers that initiate DNA synthesis (Flint et al., 2015). Primers are subsequently removed by the RNase H. In the 1980s/1990s, three prokaryotic RNases H, RNase HI, HII and HIII with roman numbers, and two eukaryotic enzymes, RNase H1 and H2 with arabic numbers, have been characterized and classified based on differences in amino acid sequences. RNase HI/H1 and retroviral RNase H are classified as type 1, whereas RNase HII/H2 are type 2 RNases H (Cerritelli and Crouch, 2009; Tadokoro and Kanaya, 2009). Furthermore, there are two yeast enzymes RNase H1 and RNase H2 that resemble the mammalian and prokaryotic enzymes (Karwan and Wintersberger, 1986). RNase H enzymes are also found in archaea (Ohtani et al., 2004). Cellular RNase H enzymes share the common activity of degrading the RNA moiety of RNA-DNA hybrids necessary, for instance, to remove RNA primers during DNA replication. Mammalian RNase H2 is composed of three subunits A, B, and C that form a functional complex with only the A subunit exhibiting enzymatic activity and the other two supplying scaffold and structural functions and mediating protein–protein interactions as well as target specificity (Crow et al., 2006). This enzyme excises ribonucleotides misincorporated in DNA molecules that can otherwise induce DNA instability and mutations. Cellular RNases H are essential in mammals and higher eukaryotes but not in lower eukaryotes and prokaryotes.

Mice deficient in RNase H1 that localizes to mitochondria die during embryogenesis, probably due to the defective processing of R-loops (Cerritelli and Crouch, 2009). R-loops are formed when an RNA strand intercalates into dsDNA, resulting in RNA-DNA hybrids and single-stranded DNA loops. R-loops affect promoter activities, with a role in gene expression (e.g., of the c-Myc proto-oncogene), genome stability, CRISPR-Cas immunity, DNA repair, and cancer formation (Sollier and Cimprich, 2015). RNases H can remove the RNA moiety and prevent deleterious DNA breaks. RNase H2 knockout mice are also not viable, and mutations in either of the human genes can cause Aicardi-Goutières Syndrome, a severe inheritable neurodevelopmental disorder (Crow et al., 2006). In this disease, uncleaved RNA-DNA hybrids accumulate within cells that possibly upregulate interferon via the nucleic acid sensor cyclic GMP-AMP synthase (cGAS) and its adaptor protein STING (Mackenzie et al., 2016). Are there retrotransposon involved? RT inhibitors are under investigation and will show!

Synthetic DNA oligonucleotides can form local hybrids and direct the RNase H to cleave the RNA moiety, which was defined as silencing by siDNA in analogy to interference by siRNA (Moelling et al., 2006; Swarts et al., 2014).

## RNase H Structure, Function and Specificity

RNase HI of *Escherichia coli* was the first one of which the three-dimensional structure was solved, revealing a conserved protein architecture, the RNase H fold (Katayanagi et al., 1990; Yang et al., 1990). RNase H folds occur in a diverse number of enzymes involved in replication, recombination, DNA repair, splicing, (retro)transposition of TEs, RNA interference (RNAi) and CRISPR-Cas immunity. Enzymes with an RNase H fold have been designated as RNase H-like superfamily (Majorek et al., 2014). RNase H folds usually contain five β-sheets (numbered 1–5) with the second being antiparallel to the other four (Ma et al., 2008) (**Figure 1B**). The catalytic core is flanked by a varying number of α-helices (A, B, C, etc.; a total of four in HIV-1 RNase H). Three to four acidic amino acid residues (aspartic acid D, or glutamic acid E) that coordinate divalent cations are required for catalysis (D443 E478 D498 D549 in HIV-1). The DEDD residues are highly conserved among type I RNase H proteins, whereas an additional C-terminal histidine (H) that is conserved in fungal, metazoan and retroviral RNases H, is replaced with an arginine (R) in archaea (Ustyantsev et al., 2015). The conserved amino acids in *E. coli* RNase H were the basis for loss-of-function mutagenesis of the HIV-1 RNase H to validate the necessary role of this enzyme for viral replication and as a drug target (Tisdale et al., 1991). RNases H act as dimers, with two Mg^2+^ or other divalent cations being essential for correct protein structure, stability and enzyme activity. Replacement of Mg^2+^ by Mn^2+^ or Co^2+^ is preferred in certain species, while Ca^2+^ ions generally inactivate catalysis (Ohtani et al., 2004).

RNase H-like proteins are directed to their nucleic acid substrates via additional factors. The best-studied example is fusion to the RT domain in retroviruses. The fusion leads to a distance of 18 nucleotides between the two active centers. An unusual structural bend is located within the extended polypurine tract (PPT) that comprises 18 nucleotides, interrupted by a CU dinucleotide. Cleavage by the RNase H domain occurs exactly at this site, generating a truncated PPT RNA primer for second strand DNA synthesis. A high degree of precision is required for this cut, leaving a terminal dinucleotide that is essential for integration and LTR formation (Sarafianos et al., 2001; Moelling, 2012).

In the case of HIV, the RNase H appears to be a processive exonuclease due to its fusion to the RT domain that pulls the RNase H during DNA synthesis. Thereby, processivity of the RNase H is pretended, even though this is an effect mediated by the RT. RNases H do not normally release mononucleotides as exonucleases would do. The catalytic activity of HIV RNase H is lower than that of the RT, and studdering of the RT enhances cleavage by the RNase H (Moelling, 2012). Exonuclease activity is controversial; however, it was recently described that RNase H-like enzymes can act as exonucleases depending on the orientation of a C-terminal alpha helix (Majorek et al., 2014). Furthermore, some RNases H can also cleave dsRNA, an activity that involves aspartic acid residues not required for cleavage of hybrid nucleic acids, as described for archaea (Ohtani et al., 2004). The cleavage of the phosphodiester bond is somewhat unusual, leading to 3′-OH, which can again be used as start for DNA synthesis, and 5′-phosphates.

## RNase H Family Members

RNases H mainly recognize structure, not sequence, and cleave one strand of double-stranded nucleic acid molecules such as RNA in DNA-RNA hybrids (RNases H), DNA in hybrids (Cas9), dsRNA (Ago/PIWI proteins of pro- and eukaryotes), and dsDNA (integrases or transposases). The importance of nucleic acid structure may be a remnant of a function in the ancient RNA world, where RNA structure was an important feature. Specificities of RNase H-like enzymes are governed by structural properties of the nucleic acid substrate, fused protein domains, protein–protein interacting factors, ion cofactors, and guide nucleic acids to find substrates in a sequence-specific manner. This, the RNases H are team players, which is supported by partnering or fused proteins that enable specific functions (**Figure 1C** and **Table 1**).

**Table 1:** RNase H-like family members and their functions.

RNHL family member	Species	Functions	Cleavage specificity
RNase H	(Para)retroviruses, ERVs, non-LTR and LTR retrotransposable elements of eukaryotes	During viral replication and retrotransposition, RNase H removes the RNA template for DNA synthesis by the RT	Endonuclease, RNA
Integrase	(Para)retroviruses, LTR retrotransposable elements	Removal of two or three nucleotides from the 3′ ends of the dsDNA copy for integration into host DNA	Endonuclease, DNA
Transposase	DNA transposons of pro- and eukaryotes	Excision of the transposon DNA from the host chromosome, for integration at a new site	Endonuclease, DNA
Terminases	DNA phages of prokaryotes	Terminases cleave the dsDNA concatemer genomes for DNA packaging and phage assembly	Endonuclease, DNA
Argonaute und Piwi-like proteins (PIWI domains)	Pro- and eukaryotes	Essential component of the RNAi immune system (siRNA, miRNA and piRNA pathways); cleavage of foreign nucleic acids, epigenetic or paragenetic silencing of TEs	Endonuclease, DNA or RNA
Cas proteins (RuvC domain)	Prokaryotes	Cas proteins for CRISPR-Cas immune system to inactivate foreign DNA of plasmids or phages	Endonuclease, DNA or RNA
RAG1	Mammals	RAG1 is a transposase, RAG1/2, are essential for V(D)J recombination, generate diversity of T and B cell receptors for antibodies	Endonuclease, DNA
Prp8	Eukaryotes	Prp8 is the “master regulator” of the spliceosome, its RNase H domain balances accuracy and efficiency of splicing	No enzymatic activity
RNase H (cellular)	Pro- and eukaryotes	Maintenance of genome stability; non-essential in prokaryotes and lower eukaryotes, but essential in higher eukaryotes	Endonuclease, RNA
RNase T	Prokaryotes	Processing of small RNAs and 23S rRNA, tRNA turnover	Exonuclease, DNA or RNA
DNA polymerases (3′ to 5′ exonuclease domains)	Pro- and eukaryotes, viruses	Proofreading during DNA replication	Exonuclease, DNA
Werner syndrome helicase (WSH) and related helicases of the RecQ family	Pro- and eukaryotes (WSH is a human protein)	Unwinding of dsDNA during repair of double strand breaks, single nucleotide damage; the RNase H domain degrades the recessed 3′ end	Exonuclease, DNA

Retroviral RNase H is discussed above. The retroviral integrase has an N-terminal zinc finger (ZNF) and a C-terminal domain (CTD), both of which mediate DNA binding to guide the catalytic core to the dsDNA substrate (Hindmarsh and Leis, 1999).

Transposases of TEs, as well as the mammalian recombination-activating gene 1 (RAG1) enzyme, also contain ZNF domains that govern dsDNA binding (Yurchenko et al., 2003). ZNF domains with desired sequence specificities can be artificially fused to an RNase H enzyme to achieve cleavage at specific target DNA sequences with potential use in gene therapy (Sulej et al., 2012). Transposases, the most abundant RNase H-like proteins (Majorek et al., 2014), mediate cut-and-paste of DNA transposons, a phenomenon originally described by McClintock (1951). Transposases cleave DNA to excise and reinsert the transposon into the host chromosome. Examples include transposases of prokaryotic transposons Tn3, Tn5 and Mu and those of the mariner/Tc3, sleeping beauty and hAT (Hobo/Activator/Tam3) families of eukaryotic transposons. Their core domains adopt an RNase H fold with catalytic DD[E/D] motif.

Terminases of phages contain an RNase H domain that cleaves dsDNA concatemers of phages into single genomes and are crucial for packaging into the virion and assembly (Feiss and Rao, 2012).

An RNase H-like fold is found in the Cas9 protein in the CRISPR-Cas9 defense system (discussed in detail below).

Argonaute (Ago) proteins mediate silencing of foreign RNAs or mRNAs via small interfering RNA (siRNA) or micro RNA (miRNA) within the RNA-induced silencing complex (RISC). Ago contain PAZ and PIWI domains that are fused and are related to the retroviral RT-RNase H fusion protein including the linker region (Song et al., 2004; Moelling et al., 2006). It is surprising, that the viral “tool kits” are similar to those of antiviral defense. Both proteins resemble each other in structure, domain organization, RNA binding and cleavage properties. The catalytic PIWI domain requires a guide RNA to localize its target nucleic acid for sequence-specific cleavage. This guide RNA is supplied by the action of Dicer, the enzyme that trims dsRNA molecules to small RNAs. Then PAZ binds the 3′-hydroxyl end of the guide RNA and directs it to the target strand (Song et al., 2004; Jinek et al., 2014). The PAZ and PIWI domains ensure correct positioning of the small RNAs to the complementary target RNA for cleavage. Ago binds to guide RNA via its PAZ pocket structural motif, originally described as primer grip for the retroviral RT-RNase H that mediates binding to the DNA opposite of the scissile RNA phosphodiester (Song et al., 2004). Depending on whether the original RNA is a double strand or hairpin-looped, the silencing is designated as siRNA using Dicer or miRNA using Drosha to process the primary pri-miRNA transcript that is further cleaved by Dicer.

Cas9 mediates the best described prokaryotic CRISPR-Cas defense system against phages and plasmids. The enzyme contains two nuclease domains and is directed to its target dsDNA by a guide RNA, a transcript originating from a previous invader that matches the DNA sequence of a new invader. It thereby creates an RNA-DNA hybrid and cleaves the invader DNA with the RuvC domain. The other one, HNH endonuclease, cleaves single-stranded DNA on the other side of the open loop. The HNH domain of the hybrid-specific enzyme contains two conserved histidines (H) and a central asparagine (N), which create a ZNF domain. The RuvC domain cleaves ssDNA, harboring an RNase H-like structure, and is split into three subdomains in the primary sequence of Cas9 (Jinek et al., 2014; Nishimasu et al., 2014) (**Figure 1C** and **Table 1**). The RuvC domain was originally identified in the RuvC resolvase that cleaves Holliday junctions, four-stranded DNA intermediates that form during recombination processes.

Remarkably, the RNase H fold is flexible enough to cleave the DNA moiety of a hybrid if a DNA phage or plasmid needs to be inactivated, whereas in the case of an RNA containing retrovirus the RNase H specifically destroys the RNA moiety of the hybrid. Thus, RNases H can specialize according to the required needs.

Poxviruses also encode a Holliday junction RNase H-like resolvase. Poxviruses are small giant viruses/*Megavirales* (described in more detail below).

Mammalian RAG1 contains a RING finger domain that is a type of ZNF domain mediating DNA binding. RAG1 is involved in V(D)J recombination during rearrangement of immunoglobulin genes and cleaves dsDNA at recombination signals via an RNase H domain with help of the RAG2 protein (Majorek et al., 2014; Moelling and Broecker, 2015). Then, V, D and J gene fragments are recombined. RAG1/RAG2 generate the diversity of T cell and B cell antigen receptors for antibodies. Both enzymes originate from a transposon and may have entered the vertebrate genomes from invertebrates such as sea urchin, sea star, and Aplysia more than 500 Mio years ago (Kapitonov and Koonin, 2015; Moelling and Broecker, 2015).

Prp8, the core protein of the eukaryotic spliceosome, has both an RNase H and an RT domain, suggesting an evolutionary origin from an ancient retroelement. Both domains have retained their three-dimensional structure and ability to bind to RNA but became enzymatically inactive (Dlakić and Mushegian, 2011). A conserved RNA recognition motif (RRM) in the RT domain is likely involved in pre-mRNA and/or small nuclear snRNA binding (Grainger and Beggs, 2005). The inactive RNase H domain may be essential for balancing accuracy and efficiency during the splicing process (Will and Lührmann, 2011; Mayerle et al., 2017). Interestingly, the RT-like domain of Prp8 likely originates from a prokaryotic group II intron (Dlakić and Mushegian, 2011), and pre-mRNA splicing probably evolved from a group II intron ribozyme (Abelson, 2013). The catalysis originally performed by a single ribozyme has evolved into the spliceosome – a large multi-component RNA-protein complex involving dozens of proteins, with Prp8 in its center.

RecQ helicases also adopt an RNase H-like fold and exert 3′-5′ exonuclease activity. RecQ domains have been identified in various DNA polymerases of prokaryotes, eukaryotes, and viruses such as phage T7 (Majorek et al., 2014). RecQ is an ATP-dependent DNA helicase implicated in diseases such as Werner syndrome (Zuo and Deutscher, 2001). The RNase H-like domain of Werner syndrome RecQ helicase has 3′-5′ exonuclease activity and degrades the recessed 3′ ends during DNA repair processes (Oshima, 2000). Mutations can lead to Werner syndrome, which is associated with accelerated aging.

Eukaryotic RNases H1/H2 require protein–protein interactions to function. The active mammalian RNase H2 enzyme is a trimer of three subunits A, B, and C, with A being the catalytically active subunit and B and C stabilizing the proper conformation of A (Crow et al., 2006). Moreover, the B subunit interacts with the proliferating cell nuclear antigen (PCNA) to bind DNA polymerase for DNA replication and repair (Chon et al., 2009). PCNA guides the RNase H2 trimer to RNA primers or misincorporated ribonucleotides to exert maintenance and repair functions (Bubeck et al., 2011).

## Abundance of RNases H

Studying evolutionarily ancient proteins and their abundance is difficult since no protein fossils are available. They were first identified by protein architecture, conserved domains, or by conserved amino acids in their catalytic centers (e.g., aspartic acid and glutamic acid in RNases H). Among the most frequent proteins in the biosphere is the RNase H – more abundant than enzymes involved in nucleotide metabolism, polymerases or kinases (Ma et al., 2008), whereby structure undergoes more stringent evolutionary constraints than does sequence (Caetano-Anollés et al., 2009).

Transposases were described as the most ubiquitous (the majority of sequenced genomes encode at least one transposase) and abundant (present in highest copy number per genome) genes in nature (Aziz et al., 2010). Ubiquitous genes are essential and indispensable in every genome, whereas abundant genes can be frequent in only a few ecosystems. Transposases are ubiquitous but also have the highest copy number per genome and may accelerate biological diversification and evolution. They carry RNase H folds, of which recently more than about 60,000 unique domains were identified based on comparative structural analyses. RNase H-like domains can be grouped by their evolutionary relationships into 152 families (Majorek et al., 2014).

## Plankton

Recently, metagenomic sequencing was performed with large sample sizes from marine samples, including small eukaryotes (protists), prokaryotes, ranging from 0.2 μm to 2 mm in size, as well as phages and viruses, obtained by the *Tara* Oceans project. According to a recent study, RTs predominate in the metagenomes (more than in metatranscriptomes), reaching up to 13.5% of the total gene abundance (Lescot et al., 2016). The authors identified about 3,000 retrotransposon/retrovirus-like RT and about 186 RNase H genes, but also 988 integrases, 556 endonucleases and helicases that add up to about 1,200 RNase H-like genes. Their weak transcriptional activity may reflect the active proliferation of retroelements that may contribute to genome evolution or adaptive processes of plankton. The RTs/RNases H are mainly found within retroelements of prokaryotes and eukaryotes. Retroelements constitute about 42% of the human genome, about 80% in maize and bread wheat, and 55% in red seaweed and many unicellular eukaryotes. Prokaryotes also harbor retroelements and DNA transposons, but less frequently than eukaryotes. About 25% of prokaryotic genomes encode at least one RT of over 1,000 different types (Simon and Zimmerly, 2008).

Sequencing of numerous genomes in the most distant organisms revealed only recently that RNase H-like molecules are among the most abundant protein entities on our planet (Simon and Zimmerly, 2008; Caetano-Anollés et al., 2009; Majorek et al., 2014). This is likely due to the fact that transposons, retrotransposons, and other retroelements are extremely abundant on our planet.

**FIGURE 2:**
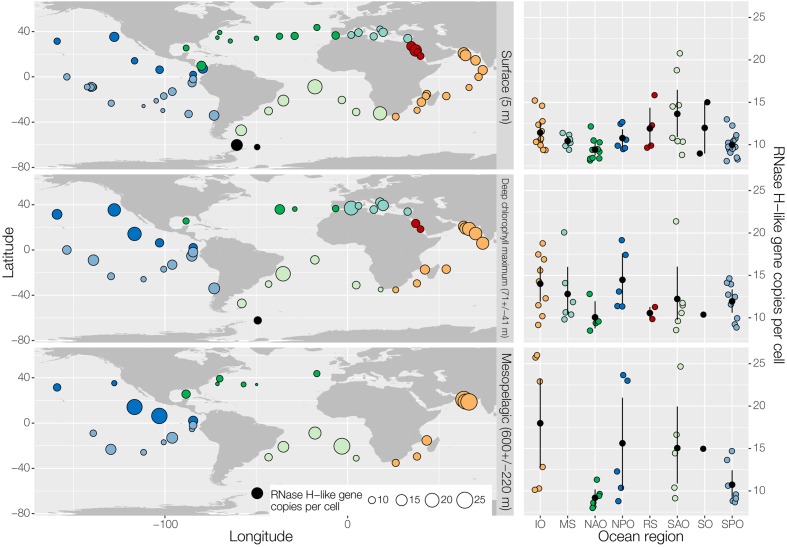
Effective abundance of RNase H-like gene family members across the global ocean. The geographic distribution of effective abundances (per cell) of all genes homologous to a set of 151 RNase H-like gene families (Majorek et al., 2014) in the ocean is shown globally (left) and regionally (right) for three different depth layers (top: surface; middle: deep chlorophyll maximum; bottom: mesopelagic). Samples correspond to prokaryote-enriched size fractions collected in the context of the TARA Oceans project (Sunagawa et al., 2015). Effective gene abundances were calculated based on annotating the Ocean Microbial Reference Gene Catalog by homology using HMM profiles of RNase H-like superfamily members (Majorek et al., 2014) with a bit score cutoff of 20. For each sample, the sum of RNAse H-like gene abundances was normalized by the median abundance of 10 universal single copy phylogenetic marker genes (Sunagawa et al., 2013) to calculate effective abundances. MS, Mediterranean Sea (light blue); RS, Red Sea (red); IO, Indian Ocean (orange); SAO, South Atlantic Ocean (light green); SO, Southern Ocean (black); SPO, South Pacific Ocean (blue); NPO, North Pacific Ocean (dark blue); NAO, North Atlantic Ocean (dark green).

The RT has previously been better characterized than the RNase H-like superfamily, hence, we were wondering about the total abundance of RNases H-like genes. For that we analyzed the RNase H gene superfamily distribution and abundance in prokaryote-enriched samples of the *Tara* Ocean (Sunagawa et al., 2015) across the global ocean at three depth layers shown globally (left) and regionally (right) (**Figure 2**). RNase H-like genes with homology to a set of 151 RNase H-like gene families were identified in all regions. On average about 10 to 15 RNase H-like gene copies per cell were detectable at all three levels. These numbers are comparable to previous findings that an average of about 13 transposase and integrase genes, the two most abundant RNase H-like genes, are present per genome, including viral, prokaryotic and eukaryotic species (Aziz et al., 2010). Thus, our data provide evidence that RNase H-like genes are probably among the most abundant gene superfamilies found in plankton organisms throughout the global oceans.

Interestingly, certain plankton populations and gene functions as judged by taxonomic marker gene sequences and gene family abundances, were dominant and shared among different regions of the oceans designated core taxa and core gene families, respectively (Sunagawa et al., 2015). Dispersal mechanisms by currents are thought to distribute these species and their genes. The less abundant species were not easily detected (Sunagawa et al., 2013). A similar phenomenon about core sequences we observed in the human gut microbiota when we analyzed the virome in comparison to the bacterial and fungal communities of a patient, who underwent a fecal microbiota stool transfer, where core sequences also dominated (Broecker et al., 2017). Thus, in the oceans, it appears that RNase H-like proteins represent important core sequences, as they were identified in all samples tested here (**Figure 2**).

## Prokaryotes

**FIGURE 3:**
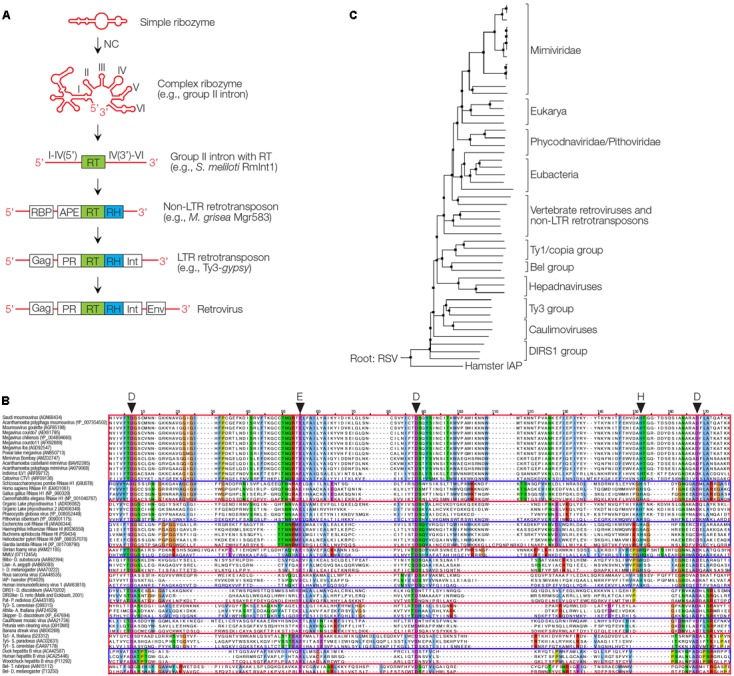
Proposed evolutionary history of the RT/RNase H and phylogeny of RNases H. **(A)** Proposed evolution from a ribozyme or viroid to group II intron, with RT, RNase H (RH) via intermediates to a retrovirus. NC is a nucleoprotein that enhances ribozyme activity and reverse transcription. APE, apurinic endonuclease; Gag, group-specific protein; Env, envelope; Int, integrase; PR, protease; RBP, RNA-binding protein. **(B)** Sequence alignment of RNases H are indicated, based on the highly conserved aminoacids D, D, E, D and partially conserved H as indicated. **(C)** Phylogenetic tree of RNase H domains found in giant viruses (*Mimiviridae*, *Phycodnaviridae*, and *Pithoviridae*), eukaryotes, various retrotransposons, retroviruses and pararetroviruses (Hepadnaviruses and Caulimoviruses). The tree is rooted to the RNase H of chicken Rous Sarcoma virus (RSV). DIRS retrotransposon found in *Dictyostelium* is the origin of many siRNAs (Boesler et al., 2014), hamster intracisternal A type particles (IAP) are transposable elements closely related to retroviruses (Qin et al., 2010).

The abundance of RTs without RNase H domains in bacteria is surprising (Simon and Zimmerly, 2008), many of them with unknown functions. It seems that host factors, precursors of RNases H or nucleases are often involved in removing RNAs instead of an attached RNase H. A number of 1021 RTs were identified in bacteria with the majority being those of group II introns (742, 73%) that encode the RT with its seven typical domains including palm and finger domains, as well as an additional endonuclease (Simon and Zimmerly, 2008). Group II self-splicing introns, a large class of mobile ribozymes, are found in all domains of life, eukaryotes, bacteria, archaea, plants, and marine plankton. An RNA loop designated as lariat RNA, and two molecules of intron-encoded protein X form an RNA-protein (RNP) complex that mediates mobility. It is site-specific and recognizes intron sequences transcribed into a DNA, targeted for target-primed RT. Group II introns may have evolved by fusion of a ribozyme with the DNA coding for the RT (Zimmerly and Semper, 2015). Group II introns are the only ones in bacteria, which are mobile (Simon and Zimmerly, 2008). Since group II introns encode an RT that lacks an RNase H domain, they require host RNases H to degrade the intron RNA template (Lambowitz and Belfort, 2015). Thus, an RNase H was required for the retromobility of the evolutionarily most ancient retroelements (**Figure 3A**).

Bacteria also harbor retrons that are related to retroelements with an unusual branched multicopy single-stranded DNA/RNA (msDNA/RNA) structure, in addition to a gene for a chromosomally encoded RT element (Inouye et al., 2011). RT partially reverse transcribes the RNA to form the branched RNA-DNA molecule by a 2′-5′ bond. This msDNA accumulates to high levels within the cell, yet its function remains elusive. The msDNA is about 3.5 billion years old. It is not mobile independently, consisting of an RT and overlapping multicopy ssDNA/RNA genes that resemble tRNA-like primers used by the retroviral RT. These structures are reminiscent of a transition from the RNA to the DNA world (Moelling and Broecker, 2015; Moelling, 2017).

Diversity-generating retroelements (DGRs) are a class of mobile elements found in phages that infect whooping cough causing *Bordetella* bacteria. *Bordetella* bacteriophage BPP-1 integrates into the bacterial genome as temperate phage and encodes an error-prone RT. The RT generates variants of the phage protein at the tip of the tail fibers of the phages. This allows for tropism changes by switching binding specificity to bacterial receptors. The number of variants is extraordinary and reminescent of V(D)J antibody diversity. DGRs are widely distributed in bacterial chromosomes, phage and plasmid genomes (Miller et al., 2008; Guo et al., 2014).

## Eukaryotes

The abundance of RT and RNase H genes in prokaryotes and unicellular eukaryotes leads to the question about their abundance in eukaryotic genomes. The human genome contains about 40,000 complete or partially truncated HERVs. HERVs are proviruses of ancient retroviral infections that have mostly degenerated to solitary LTRs (about 400,000 per genome) via homologous recombination. In addition, the human genome contains 850,000 LINEs, the long interspersed nuclear elements, including the most recent and active LINE-1 (L1) subclass, and 1,500,000 SINEs (Lander et al., 2001). Human L1 elements, of which about 100 can actively retrotranspose, encode an RT and endonuclease but no RNase H. They do not utilize RNA primers that would require an RNase H, but a target site primed mechanism for reverse transcription. Instead of degrading the RNA template for dsDNA synthesis, the LINE RT is able to displace the RNA strand during synthesis (Kurzynska-Kokorniak et al., 2007). SINEs, about 10,000 of which are active in the human genome, rely for retrotransposition on gene products of LINEs supplied *in trans*, such as the RT. Movements require endonucleases, no RNases H. Overall, almost 50% of the human genome consists of TEs, including the LINEs and SINEs and a small fraction of DNA transposons. These sequences can be regarded as a graveyard of previous infections by retroviruses and retroviral-like elements, or as a viral archive (Lander et al., 2001). Most TEs have accumulated mutations and deletions over time, rendering them inactive. Interesting, DNA transposons are rare in humans. Of all TEs, DNA transposons constitute less than 10% (over 90% being retroelements), but they are relatively more frequent in other organisms such as *Drosophila melanogaster* (over 20%). Many plants have more than 50%, *Caenorhabditis elegans* close to 90% and *Trichomonas vaginalis* almost 100% (Feschotte and Pritham, 2007).

Human ERVs and ERVs are the results of genuine retroviral infections in the past, as demonstrated by reconstitution of an infectious retrovirus named ‘Phoenix,’ obtained from alignment of near-intact HERV-K(HML-2) proviruses that first invaded the human genome about 35 Mio years ago (Dewannieux et al., 2006; Lee and Bieniasz, 2007). The Alu elements (a subclass of SINEs), which are non-coding RNAs, are somewhat reminiscent of ribozymes or viroids. The total number of retroviruses and their genes calculated from the number of 400,000 solitary LTRs would exceed the total size of the present human genome that is about 3.3 gigabases. Could it have been larger in the past as is the case in some plants? TEs are even more abundant in some plant genomes, e.g., the maize and wheat genomes contain about 80% TEs, mostly LTR retrotransposons, enlarging the total genome size to 17 gigabases in the case of wheat (Baucom et al., 2009; Brenchley et al., 2012). The large genomes may be a consequence of breeding for improving the yields for food supply. Similarly, prokaryotic genomes can contain phage sequences within CRISPR arrays, albeit in smaller quantities per genome (see below). The promoters of retroviruses or retroelements such as the LTRs can influence host gene expression, for instance, by supplying transcription factor binding sites, altering chromatin structure, or promoting regulatory non-coding RNAs both *in trans* and *in cis* (Broecker et al., 2016a). The transcription of many REs, including HERVs, is upregulated in disease states including cancer. However, whether RE activity is causal for cancer or is simply a bystander effect remains unknown. Retroviruses and REs can mobilize flanking sequences, and thereby cause gene duplication events, one of the most impactful mechanisms for creating genes with novel functions (Feschotte and Pritham, 2007).

Despite the accumulation of often deleterious mutations, TEs frequently contain functional promoters and contribute to a large fraction of the human transcriptome (Faulkner et al., 2009; Broecker et al., 2016a). TEs can influence cellular genome architecture and function by means of gene duplication or mutagenic events caused by integration (Chuong et al., 2017). While one copy can continue fulfilling acquired necessary functions, the other copy can change substantially. An interesting example of a gene duplication event is the RNase H of retroviruses, whereby the RNase H linked to the RT has deteriorated to an inactive enzyme with only linker function to the neighboring active RNase H (Ustyantsev et al., 2015).

Every retrovirus infection supplies about 10 novel genes to the cell, including those encoding the RT, RNase H and integrase. Viral integration events can lead to horizontal gene transfer (HGT) or recombination events. For example, there are about 100 retroviral oncogenes known, whereby some of them are relevant targets for human cancer therapies today such as Raf, ErbB, etc. (Flint et al., 2015).

In summary, the global abundance of RTs and RNases H can be attributed to retroelements, retrotransposons, retro- and pararetroviruses, and endogenous viruses in eukaryotic genomes. RTs are more prevalent and occur also without RNases H. There are RT groups such as prokaryotic RTs, encoded by group II introns, retrons, retrogenes, and DGRs. There are also solo RNases H without RTs, which are not the exceptions but the rule.

## Evolution of RNAse H

In the early RNA world, before proteins and DNA arose, simple self-replicating RNAs with ribozyme activity formed as primary biological entities that were non-coding (nc) but relied on structural information based on robust hairpin-looped structures. These ribozymes are capable of cleaving, joining, and evolving, as demonstrated experimentally (Lincoln and Joyce, 2009). Replication must have occurred in a prebiotic environment at hydrothermal vents down in the dark oceans with energy supply from chemical reactions without light. Ribozymes lack coding information but rely on structural information, and today are still important for the biological function of the vast majority of ncRNAs in eukaryotic cells and RNA viruses. Ribozyme-like elements act as regulatory circular or circRNAs, as so-called ‘sponges’ for small RNAs (Hansen et al., 2013). Thus, circRNAs regulate other regulatory RNAs, which may be defined as back-up or chief regulation. circRNAs may have survived until today as living fossils because of their exquisite stability (Moelling, 2012, 2017). These ancient mobile genetic elements are present until today, not only in eukaryotes but also in prokaryotes (Lambowitz and Zimmerly, 2004).

Remarkably, the ribozymes are highly related to today’s viroids, catalytically active circular RNAs. They are naked viruses, free of proteins until today. Since such elements were not considered as viruses, they were designated as viroids. Just like some other viruses the viroids can be pathogenic and inflict significant damage to many plants (Moelling, 2017). They contain a core region, which is active as siRNA for gene regulation.

Their enzymatic activity has been lost in some viroid species today – presumably in the rich cellular environment of host cells, which today harbor viroids. Gene loss as a consequence of a rich milieu is a known principle. Even in the nutrient-rich environment of the guts of an obese patient the complexity is reduced (Moelling, 2017).

Improvement of the catalytic activity of ribozymes early during evolution can be easily imagined coming from RNA binding proteins (RBPs) or small peptides with positive charge based on *in vitro* studies. Ribozymes can be stimulated enormously by the addition of RBPs, as shown for the HIV nucleocapsid (NC), leading up to a 1000-fold stimulation of ribozyme activity, which we discovered during studies to improve ribozymes for gene therapy (Müller et al., 1994).

Nucleocapsids can be detected in every RNA virus today as nucleocapsids or ribonucleoproteins (Flint et al., 2015). They are surprisingly multifunctional proteins serving many purposes. In addition to significantly enhancing the catalytic activity of ribozymes, they protect the RNA from degradation and also enhance catalysis of the RT by acting as chaperones which is almost counterintuitive but is based on the unwinding effect and the cooperativity of the multimeric NC proteins (Müller et al., 1994). NCs are rich in basic peptides such as lysine and arginine, which may have formed as smaller precursors of the NCs, and are essential components in all RNA viruses today. The NC of HIV has three such basic stretches surrounding two zinc fingers, which provide high flexibility (Müller et al., 1994) (**Figure 3A**).

Peptides could have formed in the prebiotic environment at hydrothermal vents, before the translation machinery, codons or DNA arose. The protein translation machinery must have evolved later, since ribozymes themselves contributed to the protein synthesis machinery by supplying the enzymatically active component at the center of the protein synthesis apparatus. Ribosomes today consist of about one hundred scaffold proteins for maintenance of ribosomal structure and function, and in addition some ribosomal RNAs (that serve as the basis for determination of bacterial species in microbiomes). “The ribosome is a ribozyme” is the title of an article by the Nobel Prize awardee Thomas Cech (Cech, 2000), one of the discoverers of ribozymes. Viruses with only tRNA-like structures may have contributed to the evolution of the protein synthesis machinery. Such narnaviruses exist till today in fungal species (Moelling, 2017). Also, today’s SINE or Alu sequences may fit into this concept of the predominant role of early non-coding RNAs.

Then there is a rare example of a retroviroid described in carnation plants. Carnations harbor a small viroid-like RNA, CarSV, whose homologous DNA is generated by an RT (Hegedus et al., 2001). Thus, this viroid exploits an RT, presumably provided *in trans* by a plant pararetrovirus, such as cauliflower mosaic virus.

Patel et al. (2015) recently succeeded in a “one-pot” reaction to synthesize nucleotides, fatty acids and amino acids from six elements (sulfur, nitrogen, oxygen, hydrogen, phosphorous, and carbon) in the test tube.

It is a frequent evolutionary progress and improvement that RNA leads to proteins, fulfilling similar functions but with significantly increased efficiencies. It can be easily envisaged that ribozymes became RNases H, probably by multistage processes. How the RT evolved, is still a matter of speculation. The transition from RNA to DNA must have occurred to conserve and stabilize beneficial achievements and may have led to an RT that is ubiquitously found in all coding group II introns, and whose appearance marks the beginning of the transition from the RNA to the DNA world (Lambowitz and Zimmerly, 2004; Koonin et al., 2006). Group II introns consist of highly structured RNA that developed coding capacity for an RT gene (**Figure 3A**).

An interesting intermediate between the RNA and DNA world was mentioned above, the msDNA/RNA, the retrons (Inouye et al., 2011). They appear like frozen intermediates and may be relics from early steps in evolution. They may be more ancient than the separation between prokaryotes and eukaryotes. They contain a very unusual branched rG residue covalently linking RNA and DNA. The open reading frames giving rise to msDNA/RNA are the shortest and simplest of the retroelements containing only RNase H activity in addition to the RT activity. Thus the retrons may point to the earliest possible roots of these elements and these two enzymes (Moelling, 2017).

RNase H and RT are involved in intron splicing by forming loops, the lariats. Today a eukaryotic spliceosome includes dozens of proteins. Surprisingly, Prp8 at the core of the spliceosome encodes an RT and RNase H domain, albeit both without enzymatic activities.

Group II introns have acquired an additional endonuclease activity (En) with an HNH fold. The En was likely acquired later during evolution than the RT, since no group II introns have been identified with an En in the absence of an RT (Lambowitz and Zimmerly, 2004). Of note, retrotransposition of group II introns requires host RNases H to remove the RNA template and to enable subsequent dsDNA synthesis by the RT (Smith et al., 2005). RT-encoding group II introns are likely the evolutionary precursors of non-LTR retrotransposons such as the human LINEs (Zimmerly and Semper, 2015); their RTs are highly related (Lambowitz and Belfort, 2015). LINEs no longer have ribozyme activity and encode a limited number of proteins in addition to the RT. An apurinic endonuclease (APE) cleaves DNA to initiate reverse transcription (Malik et al., 1999), replacing an RNA primer that would require digestion by an RNase H.

An evolutionary advancement of non-LTR retrotransposons, compared to group II introns, is their independence of a foreign RNase H for retrotransposition. Other non-LTR retrotransposons lacking an RNase H have evolved to an RT that displaces the RNA template during second-strand DNA synthesis (Kurzynska-Kokorniak et al., 2007). An RNase H enzyme likely evolved later than the APE and RBP (Malik et al., 1999).

Non-LTR retrotransposons evolved into LTR retrotransposons (Malik and Eickbush, 2001), the known groups being Ty1-copia, Ty3-gypsy, and BEL-Pao-like, which all encode both an RT and an RNase H (Majorek et al., 2014). Interestingly, the RNase H domain, compared to that of non-LTR retrotransposons, has lost a subdomain perhaps resulting in weaker catalytic activity (Malik and Eickbush, 2001).

An important evolutionary event was the duplication of the RNase H domain with one component leading to a tether or connection region, an inactive RNase H. This duplication is surprising for minimalistic viral genomes and may serve to fine-tune the cleavage activity of the functional RNase H by the formation of p66/p51 heterodimers found in some retroviruses, which is associated with increased rates of DNA strand transfer during reverse transcription (Ustyantsev et al., 2015). A similar ‘dual’ RNase H, active and inactive, is present in some Ty3-gypsy LTR retrotransposons (Ustyantsev et al., 2015).

The integrase exhibits an RNase H fold likely derived from the DDE transposase of a bacterial DNA transposon (Malik and Eickbush, 2001; Rice and Baker, 2001; Majorek et al., 2014). This enzyme processes the reverse transcribed dsDNA copy of the element by cleaving two to three nucleotides from the 3′ ends to expose the invariant terminal dinucleotides for insertion into host DNA (Flint et al., 2015).

The archaeal RNase H2 has been suggested to be derived from retrovirus elements (Ohtani et al., 2004).

Compared to LTR retrotransposons, retroviruses additionally gained an Envelope (Env) protein that is required for cell-to-cell transmission. Env proteins of different retrovirus lineages may have been acquired independently from different viral sources. For instance, Env of gypsy/metaviruses is likely derived from baculoviruses, dsDNA viruses of insects. The Env-derived cellular protein Syncytin contributes to syncytia formation by cell-cell fusion and originates from an endogenous retrovirus ERV-W of about 35 Mio years ago (Dewannieux et al., 2006). Due to the immunosuppressive properties of ERV-W Env, the derived syncytin prevents immune rejection of the embryo by the mother in humans and other species.

It is rather unknown that there are not only retroviruses but even retrophages in bacteria. Assuming that the RNA world preceded the DNA world one may ask whether the abundant DNA phages or any other DNA viruses had RNA or retro-precedents in ancient times. Only a few such intermediate-type viruses are known, such as the BPP-1 retrophage hosted by *B. pertussis* bacteria. This temperate phage expresses an RT whose infidelity exerts a mutagenic effect on the phage receptor gene, which can alter phage tropism. At least 36 types of such retrophages exist (Guo et al., 2014). The infidelity of the RT leads to about one mutation per thousand nucleotides and round of replication. This is a major force for change of bacterial tropism and evolution in general. Thus, these diversity generating retroelements, DGRs, demonstrate the contribution of a phage retroelement with mutagenic RT to genetic diversity and genomic variation of surface proteins of phage particles, but also of bacterial cells themselves, such as *Legionella pneumophila* (Guo et al., 2014).

One may speculate that the fast replication rate of phages and high numbers of generations may have allowed them to progress away from the RNA world and the retrophages, resulting in predominantly dsDNA phages in today’s biosphere (Moelling, 2017). The potential evolution from a non-coding ribozyme or its close relative, the viroids, to a coding one (group II intron), with the support of a NC, then to non-LTR and LTR REs and, finally, to retroviruses, is depicted in **Figure 3A**. If viroids are allowed to be defined as naked “viruses,” just lacking proteins, then the most ancient form of life was a virus, a naked viroid, a ribozyme. Then viruses would be our “oldest ancestors” (Moelling, 2012, 2013, 2017)!

## RNase H in Giant Viruses

Viruses or virus-like elements as the beginning of an RNA world have built up to bigger and more complex entities. Recently, intermediates between viruses and bacteria have attracted attention, the giant viruses or *Megavirales*. They are the biggest viruses known, surpassing the size of many bacteria and some encode genes involved in the protein translation machinery, an indicator of independent life. Are they half-finished bacteria or regressed from bacteria?

Interestingly, giant virus genomes can harbor retrotranspo sons (Maumus et al., 2014). The gvSAG AB-566-014 virus was found to encode an RT and transposase, a nuclease with an RNase H fold (Wilson et al., 2017). The virus is related to the *Cafeteria roenbergensis* (Cro) virus, a giant marine virus widespread in protists with more than 500 genes and a dsDNA genome of 730,000 nucleotides. We mined the NCBI protein and nucleotide databases for RNase H genes in the genomes of giant viruses. We thus identified a total of 17 unique RNase H proteins sequences (**Figures 3B,C**). The sequence alignment of RNase H-like enzyme sequences in comparison to known sequences with the highly conserved aminoacids D, E, D, D and the partially conserved H as indicated. The origin of the RNases H and a comparison will be subject of further analysis (Russo et al., unpublished observation).

The conserved amino acids (DEDD) are hallmarks of RNases H, and were identified in all of them, indicative of enzymatically active proteins. Giant virus RNases H stratified into two distinct clades, one containing all RNases H identified in Mimivirus-like genomes, and the other cluster containing RNases H of *Phycodnaviridae* and a *Pithoviridae*. Both clades seem to be related to eukaryotic RNases H and likely share a common ancestor, while the *Phycodnaviridae*/*Pithoviridae* RNases H are more related to eubacterial ones.

A prominent *phycodnavirus* is a green algae virus that infects *Emiliania huxleyi* coccoliths and leads to algae bloom. It also generated millions of years ago the white cliffs of Dover. We identified *Chlorella* virus sequences in the intestine of a patient after fecal transfer because of a *C. difficile* infection (Broecker et al., 2016b). No disease is known to be associated with intestinal *phycodnaviruses*.

## Viruses Protect Against Viruses

**FIGURE 4:**
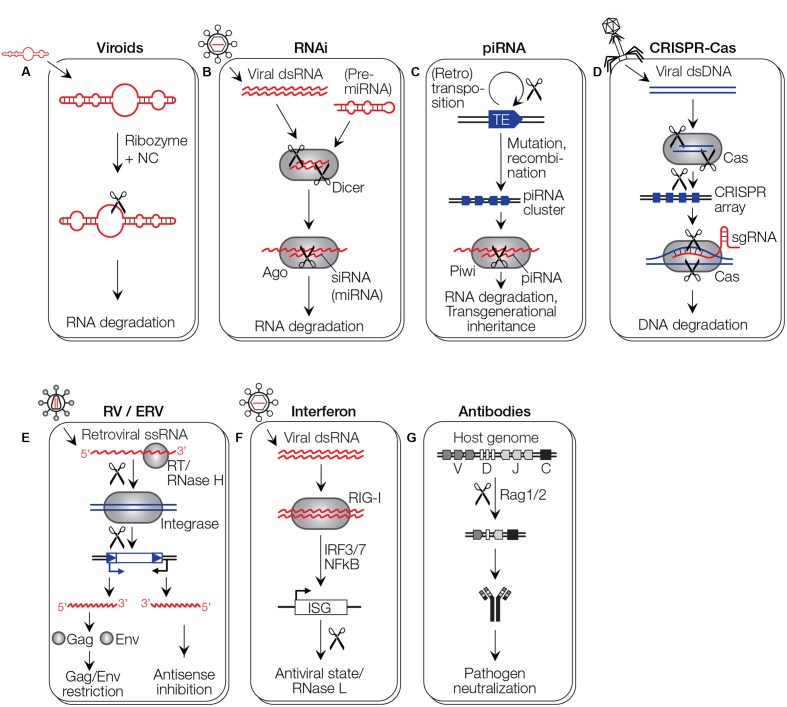
Immune system pathways. **(A)** Proposed model for an RNA-mediated immune system. A viroid enters and acts on a related viroid *in trans* by ribozyme-mediated cleavage (scissors). **(B)** RNAi requires dsRNA or pre-miRNA transcripts that are fragmented by Dicer. The resulting siRNA/miRNA acts as guide to target Ago to incoming viral RNA, whereby the nuclease domain PIWI of Ago has an RNase H fold. Whether miRNAs mediate antiviral defense is still unclear (see text for details). **(C)** In the piRNA system of animals, a clustered archive of transposable elements (TE) is generated, likely involving chromosomal rearrangement events. The piRNA cluster is transcribed in both orientations, and the resulting dsRNA is fragmented into piRNAs that target TE RNAs for cleavage by the PIWI RNase H or epigenetic repression. piRNAs are implicated in transgenerational inheritance. **(D)** In CRISPR-Cas immunity, invading DNA is fragmented by the Cas1/2 complex and fragments are integrated into CRISPR arrays. The array expresses guide RNAs that target an effector Cas9 protein to invading DNA. Two cuts are made to induce a double-strand break, one involving an RNase H-like RuvC domain, the other an HNH endonuclease domain. **(E)** Infecting retroviruses (RV) or ERVs can act against invading viruses with similar sequences when their provirus is expressed in antisense orientation by a host promoter, or a bidirectional 3′ LTR. Proteins expressed from proviruses can interfere with the infection of similar retroviruses by preventing infection through Gag-like proteins, or receptor interference through Env-like proteins. **(F)** The interferon system recognizes dsRNA molecules through RIG-I like receptors, inducing a signaling cascade that leads to the expression of interferon stimulated genes (ISGs) that induce an antiviral state in the cell. **(G)** V(D)J recombination involves the RAG1 protein with an RNase H-like domain that generates, in cooperation with the RAG2 protein, the diversity of antibodies (and T cell receptors, not shown).

Invading viruses trigger cellular antiviral responses, whereby the first virus protects the host against a second virus, at least for some time. This allows the first virus to replicate and produce progeny without competition, since resources within the cell are limited. This phenomenon is called superinfection exclusion, first described in bacteria. The viral gene products themselves once integrated into host cells can directly interfere with *de novo* infections of related viruses (Moelling et al., 2006; Moelling, 2017). Viruses can also induce cellular antiviral responses indirectly. Superinfection exclusion is found in representatives of many viral lineages, such as the positive strand ssRNA virus hepatitis C virus (Schaller et al., 2007), retroviruses including HIV (Nethe et al., 2005), small DNA viruses and the phage phiX174 (Hutchison and Sinsheimer, 1971), *Caudovirales* phages like T4 (Lu and Henning, 1994) and large DNA viruses such as *Poxviridae* (Laliberte and Moss, 2014). It appears likely that an analogous viroid/ribozyme-based superinfection exclusion system existed before the evolution of more complex viruses or cellular immune systems such as RNAi. There are different ways how viruses achieved a monopoly after entering a host cell. The strategies of viruses and antiviral responses will be discussed in below (**Figure 4**).

## Ribozymes Mediate Immunity

Ribozymes have likely been among the first biomolecules, perhaps resembling present-day ribozymes and viroids. A ribozyme/viroid may have prevented invasion by other viroids through RNA cleavage *in trans* (**Figure 4A**). This could have happened at sufficiently high concentrations even in the absence of cells, perhaps in Darwin’s famous ‘warm little pond’ or next to “Black smokers,” where chemical energy was available. Ribozymes/viroids exhibit circular hairpin loop structures, have no coding capacity but structural information only. Many of them are catalytically active until today. With the advent of cells, catalytic activity may have been lost with host cells increasingly taking over enzymatic functions. The concept that a first viroid prevents a cell from infection by a second invader, in most cases a related one, has been first described in the 1930s in *Solanaceae* plants infected with the Potato-X-Virus (Salaman, 1933). Many more examples have been demonstrated in virus-host systems of various prokaryotes, animals, plants, and humans (Folimonova, 2012; Moelling, 2017).

The cellular organism benefits from superinfection exclusion when the infecting virus is mildly pathogenic and protects against a more virulent virus. This phenomenon can be considered an early form of immune system, immunization by an ‘attenuated’ virus. Superinfection exclusion has been exploited in agricultural practice, whereby crops are infected on purpose with mild viral isolates to induce ‘cross-protection’ (Niblett et al., 1978; Folimonova, 2012). Today, viroids are exclusively found in plants, the only known exception being the viroid-like hepatitis delta virus (HDV) that infects humans. HDV is a catalytically active naked RNA virus that requires the pararetrovirus HBV to supply proteins necessary for cell to cell transmission (Taylor, 2015). Possible mechanisms of cross-protection by viroids include post-transcriptional gene silencing such as RNAi (Kovalskaya and Hammond, 2014). Although the known natural ribozymes or viroids are self-cleaving, they can be modified with relative ease to give *trans*-cleaving derivatives (Jimenez et al., 2015). Therefore, *trans*-cleaving ribozymes might also have existed or may still exist in nature.

Cleaving foreign nucleic acid molecules may be characteristic of the viroid-based immune systems while later in the DNA/protein world, molecular scissors such as RNase H-like catalytic proteins with much higher efficiencies replaced ribozymes.

## siRNA Silencing/RNA Interference

An RNAi-like defense mechanism to silence invading nucleic acids has likely evolved early during evolution, as variants of RNAi are present in all three cellular domains of cellular life (Koonin, 2017). The dsRNA of the invader is fragmented and processed to siRNA. Cell-expressed miRNA with mismatches to the target RNA is processed analogously and is used for miRNA-mediated gene silencing. The absence of RNAi in few organisms, including the yeast *S. cerevisiae*, is likely the result of gene loss (Cerutti and Casas-Mollano, 2006). RNAi, however, requires proteins, giving rise to the question how a primordial, pre-protein immune system might have looked like. siRNAs serve for antiviral defense in plants, *C. elegans*, and in many other species (Sarkies and Miska, 2014). Silencing involves RNase H-like activities found in Ago proteins for RNA cleavage and degradation. This terminates viral replication and mediates or suppresses gene expression (**Figure 4B**).

Most striking is the analogy between the antiviral siRNA system and retroviral components. We noted that PAZ-PIWI domains of Ago proteins closely resemble RT-RNase H of retroviruses. The antiviral defense system shares surprising similarities with the invading virus, including almost a dozen components of toolboxes for invasion and defense. The RT primer-grip corresponds to the PAZ pocket, DNA unwinding activity of the RT is found in the helicase domain of Dicer, the integrase generates dinucleotide overhangs with 3′-hydroxyl groups similar to Dicer, the nucleocapsid is a melting protein that protects viral RNA and may be the equivalent of the TAR RNA-binding protein, TRBP, and Fragile X Mental Retardation protein, FMRP, and the retroviral protease may be analogous to a cellular caspase (Moelling et al., 2006). Thus, a virus infection can supply the host with genes that directly become antiviral defense molecules. Retroviral infections can supply the siRNA tools for antiviral immunity.

Surprisingly enough, siRNA is not inhibiting retroviruses. A newly invading retrovirus encounters interference by several other mechanisms such as receptor blockade (see below), but not by the siRNA-based defense system. Possibly, RNAi evolved into a more efficient protein defense system against retroviruses in higher organisms. This question prompted us to analyze if antiviral siRNA activity is present in mammalian cells. Indeed, we demonstrated a weak Dicer-dependent reduction of influenza virus production by about fivefold, which was only detectable in the absence of the interferon system that dominates in mammalian cells (Matskevich and Moelling, 2007). It is surprising that the weak siRNA defense system has been preserved in mammalian cells throughout evolution (Moelling, 2013; Moelling and Broecker, 2015). It is weak in mammals, suggesting that it may be a left-over that is now overshadowed by interferons or other defense systems. RNAi mediated antiviral defense in mammals is debated by others (tenOever, 2017).

In contrast, RNAi in *C. elegans* is strong enough to efficiently prevent viral infections. siRNA is even secreted to alert neighboring worms, almost as in a larger multicellular organism. Only one virus is known to infect *C. elegans*, the Orsay virus isolated from orchards near Paris (Félix et al., 2011). Orsay virus can only be studied in worms without functional RNAi. In addition, the *C. elegans* genome harbors relatively few TEs, which may also indicate the presence of strong antiviral response. TEs and REs are among the first invading (retro)virus-like elements. In mammals, other vertebrates, and plants the RNAs of TEs are at least partially cleaved or ‘domesticated’ by the cellular antiviral defense, counteracting potentially dangerous abundant transposition by TEs, REs, SINEs, LINEs, even Alu RNA and ERVs (Roberts et al., 2014; Qin et al., 2015). This suppresses retrotransposition and supports genomic integrity (Heras et al., 2013).

## piRNas in Transgenerational Inheritance

A variation of the siRNA silencing system is the PIWI-interacting RNA (piRNA) system. It was detected by the Canadian biologist Royal Alexander Brink when he studied maize genetics, similar to Barbara McClintock about 70 years ago. They were both puzzled by the genetics of the colors of maize kernels, which did not follow the Mendelian laws of inheritance. This way McClintock discovered epigenetics and Brink paragenetics or transgenerational inheritance. Both phenomena are based on environmental influences that induce transient modifications of the genomic DNA, not stable mutations (Moelling, 2017).

As a principle in nature all epigenetic modifications of the genome accumulated by the parents are erased from the DNA in their germ cells for their offspring, so that they start with pluripotent cells with unmodified genomes. Germ line cells and stem cells have a dedicated safety mechanism that is mediated by piRNAs. They guide Piwi proteins to transcripts of REs, with the PIWI RNase H domain silencing their activity (Malone and Hannon, 2009). This is essential for fertility of the sperm. About 26–31 nucleotides in length, piRNAs are slightly larger than siRNAs or miRNAs (21–24 nucleotides) and can influence gene and TE expression by inducing epigenetic modifications such as CpG methylation in promoter regions and changes in chromatin structure (Wilson and Doudna, 2013). Especially chromatin changes can last for many generations. piRNAs exist in insects, zebrafish, and rodents, more than 50,000 unique piRNAs were detected in mice. piRNAs are transcribed from piRNA clusters and are in antisense orientation to the TEs that are targeted by the PIWI protein for degradation (**Figure 4C**). Processing of piRNAs involves the so-called ping pong amplification loop. piRNAs clusters are ‘transposon graveyards’ and therefore reminiscent of CRISPR arrays, the archives of previous infections in prokaryote genomes (Girard and Hannon, 2008; Assis and Kondrashov, 2009; Weick and Miska, 2014). A subclass of piRNAs are rasiRNAs, repeat-associated small interfering RNAs.

piRNA-mediated epigenetic modifications can be passed on to the next generation, a process called transgenerational inheritance that has been experimentally demonstrated in *C. elegans*, *D. melanogaster* and mice (Weick and Miska, 2014). It can last for up to 60 generations in *C. elegans.* The offspring worms can remember the location of a pheromone for generations even if the pheromone is not present anymore (Sarkies and Miska, 2014; Moelling, 2017). In mice, epigenetic modulation of expression of the *Kit* gene, responsible for white stripes in the tail, can be serially transmitted by small RNAs into newborn mice (Rassoulzadegan and Cuzin, 2015).

Besides restricting TEs, it has also been shown that piRNAs can exert antiviral activity and inhibit HIV and possibly other pathogens in the germ cells, preventing their vertical transmission. Silencing of TEs seems to go all the way to silencing of the complex retrovirus HIV. This is also the case in teratocarcinomas that harbor large amounts of piRNAs. Transgenerational inheritance is reminiscent of the impact of environmental influence on genetics proposed by Lamarck, a concept that has been abandoned but now experiences a revival in the form of paramutations (Moelling, 2017).

## CRISPR-Cas9

Bacteria can use the CRISPR-Cas9 defense system that is derived from previously invading phages and protects against a superinfecting new phage. This is an antiviral immune system based on the invader, where an RNase H-like molecular scissors are involved in the Cas9 protein (**Figure 4D**).

For antiviral immunity, a fragment of DNA of the first infecting phage is stored in the bacterial genome and is transcribed into messenger mRNA during a new infection. An intermediate hybrid structure is formed by the mRNA and the DNA of the newly invading phage. The DNA is cleaved by the Cas9 hybrid-specific RNase H-like activity, destroying the DNA of the new phage. The vast majority of phages have DNA genomes. In the bacterial genomes, all invading phage DNAs are stored as fragments, as an archive of the history of previous infections, the CRISPR arrays.

Interestingly, in the case of RNA phages such as MS2, a relative of Cas enzymes of a different class (Cas13a, formerly C2c2) exhibits CRISPR-Cas-like RNA-guided RNase H-like activity, as required to inactivate an RNA phage genome (Abudayyeh et al., 2016). Other Cas-related systems exist that act not only for spacer acquisition for adaptation, but also expression, target cleavage by interference, and regulatory functions (Nuñez et al., 2015; Silas et al., 2016). In addition to the numerous Cas systems a Cas10 cooperating with a ribonuclease has been described which not only destroys the DNA of the invader but also the RNA (Kazlauskiene et al., 2017), possibly improving the defense efficiency by a “back-up.”

The defense strategies of CRISPR and siRNA are related, one difference lies in the storage of the genetic information of the invader. Double-stranded RNA of siRNA cannot be integrated into the host DNA as dsDNA but is either degraded or transiently stored within the proteins of RISC, but not for a new generation.

The CRISPR-Cas system most likely originated from a class of DNA transposons called casposons that rely on Cas-like activity to spread throughout prokaryotic genomes (Krupovic et al., 2017). Casposons may have contributed to the distribution of CRISPR-Cas defense system in archaea and bacteria.

It is often stressed that the CRISPR-Cas9 system is the only inheritable immune system, which does not exist in any other organism or host. One may contradict if the endogenous retroviruses are considered. They were once exogenous viruses and were endogenized and passed on for generations. Some of them can protect against superinfection by the same or related viruses. This is an inherited defense system, also reflecting the history of previous infections just like the archive described for CRISPR inserts. Archives of previous infections, fossil records, are represented by endogenous retroviruses. This has most surprisingly been demonstrated by the resurrected ERVs, which after 35 Mio years could be “repaired” from defective retrovirus inserts to an infectious one, designated as Phoenix (Dewannieux et al., 2006; Lee and Bieniasz, 2007).

What amount of phage DNA can be accumulated inside a bacterial genome? Can they become 35 Mio old or even older, as HERVs do? Can the archives be deleted, how and when? Can we learn a lesson from the ERVs? Could there be something similar to the removal of ERVs or HERVs, where shorter versions and finally solitary LTRs are left as minimal footprints? Is there something similar for phage genomes? What would minimal phage footprints look like? Are the direct repeats (DRs) with or without spacer sequences candidates resembling LTRs?

## Superinfection Exclusion by Retroviruses and ERVs

Retroviral infections in a mammalian or other cell types can lead to antiviral resistance due to receptor interference, which is based on the expression of a retroviral gene product, which binds to or downregulates the cell receptor for virus uptake and prevents *de novo* infection. This mechanism of superinfection exclusion resembles the effect of interference, the interfering consequence of the first viral infection against the next one. A basic viral principle can be assumed to be involved in this shut-off, the limited resources inside a cell for several simultaneous virus replications.

Retroviral endogenization and protection from superinfection was recently shown in koalas. Koalas were transferred as endangered species (by car accidents!) to an island next to the Australian mainland to be protected from going extinct, where they attracted Gibbon Ape Leukemia virus, GALV, infections and died of leukemia (Tarlinton et al., 2006). Within 100 years an antiviral resistance and survival developed as a consequence of endogenization of the retrovirus. The establishment of endogenization of a virus as the cause of resistance against infections of the same type is a more general mechanism, also known in the formation of resistance in bats against a variety of viruses (Wang et al., 2011). The gene products of the first virus in chimpanzees protects them against a novel infection (Tarlinton et al., 2006; Denner and Young, 2013) (**Figure 4E**).

Could HIV be able to endogenize and then prevent exogenous infections? For endogenization, the virus needs to infect germline cells. A recent report describes this possibility. But what required 100 years in koalas in about 20 generations would possibly require 300 to 500 years in humans (Moelling, 2017).

Also in honey bees, an endogenous Israeli Acute Paralysis Virus is known to protect against related viruses (Aswad and Katzourakis, 2012). Furthermore, Borna viruses that are replicating as well as being in the process of endogenization, may cause resistance. Also, Ebola-, Bunya- and Hantavirus-related sequences have been identified in vertebrate genomes and may protect the organisms against infection of the same virus. These viruses are single-stranded RNA viruses that would normally not integrate. Yet they were determined as endogenous viruses, indicating illegitimate reverse transcription and DNA integration. They must have been reverse transcribed by a foreign RT of LINE or other retroviral elements (Belyi et al., 2010a). In total, 10 types of incoming non-retroviral RNAs must have been illegitimately reverse transcribed and integrated into host genomes (Belyi et al., 2010a,b; Aswad and Katzourakis, 2012). Endogenous Bornavirus sequences express not only viral NC proteins in human cells but also polymerase and glycoproteins, which may protect humans from infection, conferring immunity against related viruses. In contrast, horses that lack endogenous Borna viruses, more frequently suffer from Borna disease that includes symptoms of depression. DNA viruses such as circoviruses may show similar modes of protection (Belyi et al., 2010a). Such viral archives also exist in prophages, where fragments of phage and plasmid DNA are integrated and inherited as spacers present in CRISPR arrays.

As mentioned, syncytins show sequence homology to the transmembrane protein gp41 of extant retroviruses as well as HIV. The transmembrane protein causes immune suppression in the mother to prevent an immunological rejection of her embryo. Syncytins are related to the retroviral Env proteins causing immune suppression there also. This is one of the most surprising examples of how a retrovirus shaped the human genome. The effect is also observed in other mammalian species such as cows and the syncytin genes have been acquired by independent retroviral endogenization events in different mammalian lineages (Imakawa et al., 2015). There are many host restriction factors against retroviruses developed by the hosts, many of them are not of direct viral origin but are cellular antiviral factors.

We have analyzed a HERV family member belonging to the mouse mammary tumor virus related family HERV-K (HML-10) (Broecker et al., 2016a). It has integrated into the human genome about 35 Mio years ago. We demonstrated that one of the HERVs expressed a transcript in antisense orientation to a transcript of a cellular pro-apoptotic gene, resulting in antisense inhibition of an apoptotic gene leading to cell survival and a malignant phenotype (**Figure 4E**). It is surprising that suppression of apoptosis was detected with HERV, because cell survival guarantees higher viral progeny, yet there is no progeny, as the open reading frames of this HERV have been inactivated by mutations. The anti-apoptotic effect may therefore be a relic from former days of replication competence 35 Mio years ago. The LTR promoters of this HERV family are cytokine-regulated and highly variable with respect to orientation and expression levels, making it difficult to predict something about their general role in human cancer or other diseases. Recently, the Env protein of HERV-K was shown to inhibit HIV infection *in vitro*, which suggests that superinfection exclusion does not even affect the identical species only but also others if related enough (Terry et al., 2017).

Superinfection exclusion mediated by the expression of ERVs may constitute a simple form of inheritable immune system in eukaryotes. An antisense transcript of the ERV can be generated, for instance, if the ERV integrates in opposite orientation into the intron of a host gene. Indeed, the opposite orientation is usually favored for HERVs that integrate into introns. We and others have shown that HERV-originating transcripts that are opposite to intron sequences, can downregulate the expression of host genes *in cis*, and suppression in *trans* may also occur (Gosenca et al., 2012; Broecker et al., 2016a). Similarly, transcription of cellular genes that contain intronic ERVs in opposite orientation will generate retroviral antisense transcripts that might protect against exogenous infections (Mack et al., 2004). Such an inhibitory mechanism by long non-coding RNAs may not require the RNAi machinery, since in the RNAi-deficient *S. cerevisiae*, Ty1 LTR retrotransposons are suppressed *in trans* by lncRNAs originating from antisense promoters within Ty1 elements (Harrison et al., 2009).

Endogenous retrovirus-mediated superinfection exclusion can also be achieved through expression of retroviral proteins. In mice, a genetic factor puzzled retrovirologists for decades, the Fv1 in mice, which leads to genetic resistance against retrovirus infections and is associated with expression of a Gag-like protein of a mouse retrovirus. Similarly, Fv4 expresses an Env-like protein (Aswad and Katzourakis, 2012). Fv1 likely interacts with the pre-integration complex of MuLV, preventing genomic integration, while Fv4 acts via receptor interference to inhibit viral entry (**Figure 4E**). Receptor internalization is another mode of defense to prevent entry by a competitor, which is sometimes only transient until the cell has recovered.

Thus, retroviruses protect a host cell from another retrovirus by viral gene products, not the components of the siRNA system.

## Protein-Based Defense: Secreted Interferon

The interferon (IFN) system is a form of protein-based immune defense with an orthologous signaling pathway as the siRNA system (**Figure 4F**). It is well accepted that the RNA world preceded the protein world and the IFN system may have been a later achievement during evolution. The most striking similarity is the secretory mechanism of IFN reminiscent of siRNA, both of which are secreted from an infected cell and warn the uninfected neighboring cells by stimulating their defense system, at the expense of the primary cell that dies. Three responses can be distinguished in either the siRNA or IFN system: silencing, mRNA degradation, and inhibition of translation (Moelling and Broecker, 2015). First, gene deamination in the IFN system correlates with methylation of chromatin by repetitive associated silencing rasiRNA. Secondly, dsRNA is detected by oligoadenylate synthetase (OAS), causing synthesis of 2′-5′ oligoadenylates that in turn activate RNase L for viral mRNA degradation by the IFN system, equivalent to mRNA degradation by siRNA or miRNA, whereby the miRNA system also leads to inhibition of translation. Thirdly, the protein kinase R (PKR) is activated through double-stranded RNA binding, which induces autophosphorylation, leading to activation of its kinase activity PKR. This then inactivates translation initiation factor eIF2a by phosphorylation, leading to inhibition of translation in the interferon system. More details have been published previously (Moelling and Broecker, 2015).

The immune systems are related, one based on nucleic acids, the other one on proteins. The innate IFN system in eukaryotes is based on proteins and is sequence independent. However, its mechanism closely resembles the sequence specific siRNA system in most steps. It appears that the RNA has evolved toward a protein-based mechanism in an orthologous fashion with similar steps to fulfill similar functions.

The IFN system recognizes dsRNA molecules through RIG-I like receptors, inducing a signaling cascade that leads to the expression of interferon-stimulated genes (ISGs) that induce an antiviral state in the cell. RIG-I is structurally related to Dicer, and similar to small RNAs in plants and *C. elegans*, IFNs are secreted. Although no RNase H molecule is involved in IFN signaling, there are other RNases and striking similarities between RNAi and IFN signaling, most strikingly the secretion and warning of neighboring cells (Moelling and Broecker, 2015).

## Antibody Diversity Generated by RAG1

Another protein-based immune system is constituted by antibodies. The diversity of populations of immunoglobulins and T cell receptors is generated in many species by V(D)J recombination, by combinatorial joining of segments of coding sequences. V(D)J recombinations can lead to millions of different functional immunoglobulin and T cell receptor genes. This recombination is mediated by the RAG1 recombinase protein with an RNase H-like domain with a zinc-binding catalytic center with the conserved D, D, E, similar to transposases. The catalytic activity of RAG1 is supported by complexing to the RAG2 protein, which may also be derived from transposases but is enzymatically inactive (**Figure 4G**). The high degree of sequence diversity required for antibody-based defense is generated by the transposon-type cut-and-paste mechanisms. The RNase H-like cleavage activity cleaves and performs an additional step by closing the DNA to hairpins and releasing excised circles. V(D)J recombinations evolved from transposons and possibly exhibited RNase H-like enzyme activities in the 900 Mio years old immune system. This transposon-like diversification system of our immune system was also described in the house fly, which, however, has no adaptive immune system. Its name is Hermes and it is very mobile for innovation in the fly genome (Moelling and Broecker, 2015). It is a remarkable ‘altruism’ that siRNAs in plants, *C. elegans*, IFNs, and the antibodies are secreted from an infected endangered cell to protect other cells.

One can classify antiviral systems by stating that invading RNA is counteracted by siRNA while invading DNA is defended by the CRISPR-Cas systems in bacteria. But other mechanisms: there are many more defense mechanisms in virus-infected cells, not only viral-coded gene products against viruses but also numerous cellular restriction “factors.”

## Conclusion

We are describing the importance and wide-spread distribution of the RNase H-like family members. They are among the most abundant molecules on our planet and present in all forms of life. This study contributes the identification of highly conserved RNase H-like proteins in a variety of marine samples. Indeed, also the RT and related other nucleic acid synthesizing enzymes are similarly abundant. Whenever nucleic acids are synthesized, also removal mechanisms must exist. Thus, RTs and RNases H may have cooperated throughout evolution. With RNA as the primary molecule in evolution it is not surprising, that RNA-degrading molecules arose for defense, ranging from ribozymes/viroids to RNases H. The role of RNases H-like molecules in antiviral defense was stressed here, among others by the surprising evidences in an earlier study, that the components of retroviruses and siRNA-mediated antiviral defense are orthologs, similar in structure and function.

The origin of the protein enzymes is not known, yet one might conceive that catalytic RNAs such as ribozymes and the viroids may be the RNA precursors to RNase H-related proteins. They are of universal usefulness due to their lack of specificity. This is compensated for by being a team-player with other factors, such as RNAs or proteins, to gain specificity. The RT may have evolved from simpler polymerizing structures, which may have some evolutionary connection to the rather unexplored msRNA/DNA elements. Retroelements are among the oldest ones as drivers of evolution and genome diversity. The relationship between so diverse species as bacteria and mammals was stressed here by comparing the CRISPR-spacers and the HERV sequences in the genomes as archives from earlier phage and viral infections.

Understanding the role of PIWI/RNase H in transgenerational inheritance will be fascinating.

## Author Contributions

KM has conducted research on the RNase H for 50 years, discovered the retroviral RNase H, drafted the concept of this article and wrote the majority of the text. FB has 10 years of experience in the research field, designed most of the figures and made significant contributions to the concept, bioinformatics and text. GR performed multiple sequence alignments and generated the phylogenetic tree of RNase H proteins in **Figure 3** (unpublished). SS is part of the TARA Oceans project and generated the data and figure presented in **Figure 2** (unpublished). All authors read and approved the final version of the manuscript.

## Conflict of Interest Statement

The authors declare that the research was conducted in the absence of any commercial or financial relationships that could be construed as a potential conflict of interest.
